# Reliability and Validity of a Flume-Based Maximal Oxygen Uptake Swimming Test

**DOI:** 10.3390/sports11020042

**Published:** 2023-02-08

**Authors:** Elizabeth F. Nagle, Takashi Nagai, Anne Beethe, Mita Lovalekar, Meghan S. Tuite, Meaghan E. Beckner, Jacquelyn N. Zera, Mary E. Sanders, Chris Connaboy, John P. Abt, Kim Beals, Scott M. Lephart, Robert J. Robertson, Bradley C. Nindl

**Affiliations:** 1 Department of Health and Human Development, University of Pittsburgh, Pittsburgh, PA 15261, USA; 2 U.S. Army Research Institute of Environmental Medicine, Natick, MA 01760, USA; 3 Kinesiology and Health Science, Utah State University, Logan, UT 84322, USA; 4 Department of Sports Medicine and Nutrition, University of Pittsburgh, Pittsburgh, PA 15213, USA; 5 Department of Exercise Science, John Carroll University, Cleveland, OH 44118, USA; 6 Reno School of Medicine & Community Health Sciences, University of Nevada, Reno, NV 89557, USA; 7 Center for Lower Extremity Ambulatory Research (CLEAR), Rosalind Franklin University, North Chicago, IL 60064, USA; 8 Children’s Health Andrews Institute for Orthopaedics & Sports Medicine, Plano, TX 75024, USA; 9 School of Medicine, University of Kentucky, Lexington, KY 40536, USA

**Keywords:** VO_2_max*_sw_*, swimming flume protocol, freestyle performance swims, swimming performance

## Abstract

A mode-specific swimming protocol to assess maximal aerobic uptake (VO_2_max*_sw_*) is vital to accurately evaluate swimming performance. A need exists for reliable and valid swimming protocols that assess VO_2_max*_sw_* in a flume environment. The purpose was to assess: (a) reliability and (b) “performance” validity of a VO_2_max*_sw_* flume protocol using the 457-m freestyle pool performance swim (PS) test as the criterion. Nineteen males (*n* = 9) and females (*n* = 10) (age, 28.5 ± 8.3 years.; height, 174.7 ± 8.2 cm; mass, 72.9 ± 12.5 kg; %body fat, 21.4 ± 5.9) performed two flume VO_2_max*_sw_* tests (VO_2_max*_sw_*A and VO_2_max*_sw_*B) and one PS test [457 m (469.4 ± 94.7 s)]. For test–retest reliability (Trials A vs. B), moderately strong relationships were established for VO_2_max*_sw_* (mL·kg^−1^·min^−1^)(r= 0.628, *p* = 0.002), O_2_pulse (mL O_2_·beat^−1^)(r = 0.502, *p* = 0.014), VEmax (L·min^−1^) (r = 0.671, *p* = 0.001), final test time (sec) (0.608, *p* = 0.004), and immediate post-test blood lactate (IPE (BLa)) (0.716, *p* = 0.001). For performance validity, moderately strong relationships (*p* < 0.05) were found between VO_2_max*_sw_*A (r =−0.648, *p* = 0.005), O_2_pulse (r= −0.623, *p* = 0.008), VEmax (r = −0.509 *p* = 0.037), and 457-m swim times. The swimming flume protocol examined is a reliable and valid assessment of VO_2_max*_sw.,_* and offers an alternative for military, open water, or those seeking complementary forms of training to improve swimming performance.

## 1. Introduction

Competitive, fitness, and recreational swimming have grown in popularity with an increase in participation over the last 5 years [1,2]. The current areas of study among sport scientists and coaches dedicated to improving swimming performance include determinants of aerobic and anaerobic energy demands and/or capacity, in addition to a further understanding of fluid dynamics and associated drag [3,4,5]. A pool environment remains the most practical setting to examine these factors. Today’s swimming flumes are manufactured for commercial, residential, clinical hydrotherapy, and plunge pool usage. Furthermore, swimming flumes provide additional opportunities through the quantification of workloads for the advanced study of factors related to swimming performance. Furthermore, they provide opportunities to explore the development of accurate testing methodologies and novel training practices [3,6,7,8].

A swimming flume directs water circulation through a controlled closed loop system where a “test basin” receives laminar and constant water flow, enabling a swimmer to remain stationary at velocities controlled by the investigator [3]. By controlling for velocity, depth, and water temperature, a swimming flume can provide a standardized and more controlled testing environment [3,9]. Early research paradigms used swimming flumes to study swimming mechanics with less focus on swimming physiology [3]. However, more recent advanced studies have employed flume-based tests to both energetics and stroke mechanics in a temporal fashion [10,11,12]. A continuous graded swimming flume test protocol can assess cardiorespiratory and metabolic factors that determine maximal oxygen uptake, energy expenditure, as well as the economy of a swimmer’s stroke (i.e., stroke efficiency) [8,12,13].

Evidence to support validity of a maximal oxygen uptake test protocol in a swimming flume as it relates to actual pool-based performance is limited. This may be due, in part, to a lack of accessibility to a swimming flume, as well as the technical challenges associated with indirect calorimetry measurement in water [14]. Studies have used swimming flume protocols to examine relationships between maximal oxygen uptake (VO_2_max*_sw_*; mL·kg^−1^·min^−1^) and other variables such as critical velocity to explore flume and swimming performance with mixed results. This was due, in part, to a lack of a standardized swimming protocol, as well as the criterion swimming distances chosen [8,9,15,16,17,18]. Despite its potential for test standardization, investigators have questioned a swimming flume’s utility and predictive validity due to a lack of uniform water flow and its effect on drag detected within the test basin [19]. These conditions may negatively influence stroke mechanics or ability to achieve a maximal swimming velocity and add concern for an ability to accurately measure physiological outcomes throughout a swimming flume protocol [20,21].To date, no study has examined both the reliability and validity of VO_2_max*_sw_* using a continuous maximal oxygen uptake swimming flume test protocol that measures its relationship to swimming performance. A valid and reliable flume protocol could provide coaches and trainers with a better understanding of the bioenergetic demands of a swimming event greater than five minutes and, similarly, measure the estimated energy cost of submaximal swimming velocities. Therefore, the purpose of the present investigation was to determine the reliability and validity of a continuous graded intensity swimming flume protocol to measure VO_2_max*_sw_* in a mixed sample of health-fitness and competitive masters swimmers. Understanding the metabolic cost of swimming could assist with individualized programs that target the most critical physiological objectives to improve performance and/or health.

## 2. Materials and Methods

### 2.1. Experimental Approach

A multiple observation, within subject, counter-balanced design was employed. Subjects were familiarized with the experimental trials via an orientation session. On separate days, two continuous swimming flume maximal aerobic uptake trials (VO_2_max*_sw_*A and VO_2_max_sw_B) and a criterion measure 457-m swimming trial were administered. The three experimental trials were separated by a minimum of two but not more than seven days.

### 2.2. Participants

Nineteen male (*n* = 9) and female (*n* = 10) participants (mean +/− SD; 28.5 +/− 8.3 years) were recruited from Pittsburgh and surrounding communities. The descriptive characteristics of the participants are presented in Table 1. Participants were included if they satisfied the following criteria: (1) 18–45 years old; (2) comfortable swimming in shallow water; (3) intermediate level swimmer or higher defined as able to complete a 182-m freestyle swim using rhythmic breathing in under 4 min; and (4) currently physically active. Following initial communication, potential participants were screened using a medical history and physical activity readiness questionnaire (PAR-Q) [22]. If eligible, participants were notified of benefits and risks, provided consent, and were scheduled for the orientation. All procedures were approved by the University of Pittsburgh Institutional Review Board.

### 2.3. Procedures

Upon laboratory arrival, standing height (cm) was measured, and body composition was assessed using air displacement plethysmography (Bod Pod; Cosmed, Chicago, IL, USA) [23]. Swimming flume tests were conducted using the SwimEx swimming flume (5.96 m long × 6.11 m wide × 1.76 m deep) (Warren, RI, Model SwimEx 600T2) with velocities ranging from zero to approximately 1.51 m∙s^−1^. The pool test was conducted in the University of Pittsburgh’s indoor pool at a depth of 1.3 m with a measured performance distance of 22.9 m in length. The pool temperature was maintained at 27.5 °C for swimming flume and pool trial. The purpose of the orientation trial was to control for test familiarization bias that may have transpired in participants who had not previously undergone a VO_2_max*_sw_* test in a swimming flume environment.

For the orientation trial, subjects were provided a description of the test protocol that included a video depicting the flume-based VO_2_max*_sw_* test. Participants were fitted with a telemetry heart rate (HR) monitor (Kempele, Finland) and Cosmed Aquatrainer (Rome, Italy) (i.e., respiratory mouthpiece and nose clip) to become familiar with test procedures. Participants were encouraged to experience swimming at different velocities by self-selecting three continuous 2-m stage velocities perceived to represent low (50%), moderate (70%), and high (90%) intensity efforts. In order to establish speed for the initial stage of the experimental trial, a flume velocity that corresponded to 85% of the subject’s age predicted maximum heart rate was identified [17]. Expired ventilatory volume (VE) (L∙min^−1^; standard conditions of temperature, pressure, and dry (STPD)) and respiratory O_2_ consumption (L∙min^−1^) and CO_2_ (L∙min^−1^) produced were determined by 15-s intervals using the Cosmed K4b and Aquatrainer (Rome, Italy) respiratory-metabolic unit throughout the orientation and subsequent experimental trials. The portable Aquatrainer and metabolic unit were suspended in a position that required a stationary freestyle stroke while providing an in-flume, continuous measure of VO_2_ and respiratory exchange ratio (RER). To assist with maintaining a stationary body position, underwater light sensors signaled participants to remain in the same position relative to the length and width of the flume throughout each stage. Subjects were also habituated to the Aquatic OMNI (0–10) rating of perceived exertion (RPE) scale. The OMNI scale uses verbal, numerical, and pictorial categories representing equal intensity intervals with zero being extremely easy and 10 being extremely hard. The OMNI Scale is used by exercise physiologists and coaches to objectively evaluate an individual’s perceived level of effort, strain, discomfort, and fatigue felt during aerobic or resistance exercise [24,25].

A standardized series of OMNI scale rating guidelines and procedures were explained during orientation to assist with self-selection of velocities that corresponded to specific intensities. The OMNI scale was also used as a post-test rating of effort immediately following all VO_2_max*_sw_* flume tests and the performance swim trial.

Within one week, VO_2_max*_sw_* trials were administered. First, participants were fitted with the telemetry heart rate monitor, facemask, and mouthpiece described previously. Subjects swam in a stationary position against progressively increasing flow rates in the SwimEx flume [17]. The continuous graded swim test protocol began with a two-minute stage at a flow rate that produced 85% of the participant’s age predicted maximal heart rate (220-participant’s age), i.e., as observed during the orientation swim. Following the initial two-minute Stage 1, each succeeding test stage was 30 s in duration. Beginning with Stage 2, flume velocity increased by approximately 0.09 m·s^−1^ at the beginning of each stage until the participant achieved VO_2_max_sw_ or was unable to maintain a stationary position due to fatigue. VO_2_ max_sw_ was identified as a change in VO_2_ of <2.1 mL∙kg^−1^∙min^−1^ with increased exercise intensity and/or the highest VO_2_ reached at maximal swimming intensity. Secondary VO_2_max*_sw_* test criteria included one or more of the following: 1) a respiratory exchange ratio (RER) > 1.10 (defined as ratio of (CO_2_): (O_2_)); (2) HR ± 5 b·min^−1^ of the age-predicted maximum; (3) a RPE (OMNI scale) > 9; (4) volitional test termination due to exhaustion, and (5) blood lactate > 8.0 mmol∙L^−1^ [13,26,27]. When the test was terminated (i.e., unable to maintain stationary position in the swimming flume), the participant lifted their head out of the water, whereby investigators immediately stopped the swimming flume and removed the mouthpiece and nose clip.

To determine secondary VO_2_max*_sw_* test criteria, immediate post-exercise blood lactate (IPE (BLa)), immediate post-exercise RPE (IPE-RPE), HR, and RER were measured for each VO_2_max*_sw_* experimental trial. A 5μL plasma lactate capillary sample was obtained prior to warming-up and immediately post-test for the two experimental swimming flume trials and performance swim using a Lactate Pro (Arkray Inc., Kyoto, Japan) monitor. After the metabolic equipment was removed, subjects completed a cool-down by swimming for three minutes at a low intensity flume velocity or until heart rate decreased to <110 b∙min^−1^. Experimental Trial B procedures were identical to those for Trial A.

Within seven days, subjects completed the 457-m freestyle performance swim in a 22.86-m pool. Immediately prior to the performance swim, resting heart rate and blood lactate measures were obtained. Next, a swimming warm-up distance of 250–450 m of freestyle swimming at self-selected speeds began at 50% and gradually increased to 80–90% of maximal velocity. Following a rest period of 2–3 min, subjects were instructed to perform the swimming distance at 100% of their maximal velocity. Performance time was assessed using an Accusplit digital stopwatch (Pleasanton, CA, USA) and documented to the nearest 100th of a second. Immediate post-exercise (IPE) measure of post-exercise blood lactate (IPE [BLa]), heart rate, and RPE were obtained following the performance trial. The cool-down at an ad libitum swimming pace occurred until recovery HR was to <110 beat∙min^−1^.

### 2.4. Statistical Analysis

A priori sample size calculation showed that 19 subjects would result in at least 80% power at a 0.05 significance. Descriptive statistics (mean ± standard deviation) were calculated. Shapiro–Wilk tests were conducted for normality. Significance was set a priori at *p* < 0.05, two-sided. The test–retest reliability of the flume-based variables was assessed with an intraclass correlation (ICC (2,1)). A paired *t*-test was also conducted in order to examine differences between trials. The validity of the flume-based tests was assessed using Pearson or Spearman correlation coefficients as appropriate. VO_2_max*_sw_* A trial data was only used to simulate application where little to no practice/orientation would typically occur for a participant prior to a swimming flume trial. Bland–Altman plots were graphed to assess concordance between VO_2_max*_sw_* A and B trials including systematic bias, patterns of error, and 95% limits of agreement [28].

## 3. Results

### 3.1. VO_2_max_sw_ Protocol: Test Reliability

Nineteen participants finished the VO_2_max*_sw_*A and VO_2_max*_sw_*B trials. The test–retest reliability results for physiological responses measured during the VO_2_max*_sw_*A and VO_2_ max*_sw_*B flume trials are presented in Table 2.

This sample was comprised of an approximately equal number of men and women and represented a distribution of the general population. Therefore, findings are reflected as a mixed sample of males and females to better reflect the general population. ICCs revealed moderate to strong reliability for VO_2_max*_sw_* (ICC = 0.628, *p* = 0.002), O_2_pulse (ICC = 0.502; *p* = 0.014), VEmax (ICC = 0.671; *p* < 0.001), IPE-RPE (ICC = 0.539; *p* < 0.006), RERmax (ICC = 0.559; *p* < 0.002), IPE [BLa] (ICC = 0.716; *p* = 0.001), and final test time (ICC = 0.608; *p* < 0.004) determined by the two swimming flume experimental trials (Table 3). In addition, VO_2_ (mL∙kg^−1^∙min^−1^) measures were reliable for Stages 2 through 6 (ICC = 0.465 to 0.669); *p* < 0.05). 

**Figure 1:**
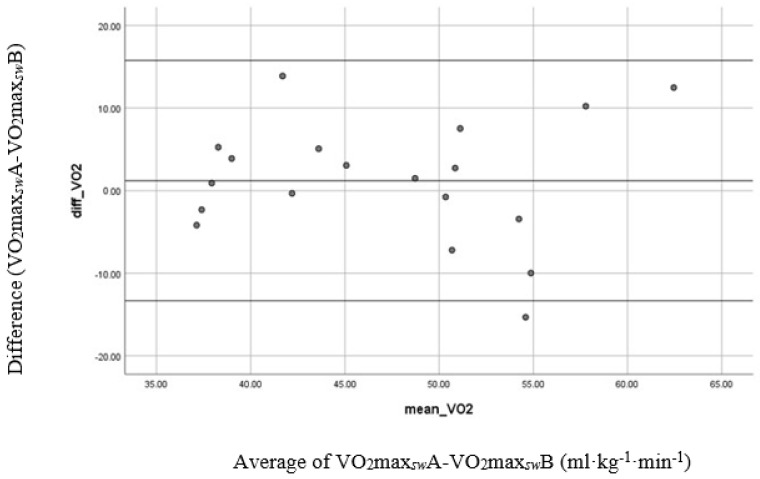
Bland–Altman plot for VO_2_max*sw* test reliability. The difference between VO_2_max*sw*A and VO_2_max*sw*B is shown on the y-axis. The gray line shows the mean of the differences.

The paired *t*-test showed no significant differences (*p* > 0.05) between Trials A and B for physiological and perceptual variables except for RERmax and IPE-RPE (*p* < 0.05). Bland–Altman analyses showed that the VO_2_max*_sw_* tests reflected a systematic error with a slightly positive mean bias (1.21 mL∙kg^−1^∙min^−1^), which specifies that participants had a slightly higher value during test 2 as compared to test 1; however, this difference did not demonstrate statistical significance. The 95% limits of agreement were (−13.34 mL∙kg^−1^∙min^−1^; 15.76 mL∙kg^−1^∙min^−1^), suggesting moderate reliability (Figure 1).

### 3.2. VO_2_max_sw_ Protocol: Validity

Validity of the VO_2_max*_sw_* test’s physiological responses were correlated to the 457 m PS time (Table 4). The participant completed both VO_2_max*_sw_*A and PS time trials. Moderately strong negative correlations were found between the 457-m PS time and VO_2_max*_sw_*A (r = −0.648; *p* = 0.005), as well as O_2_pulse (r = −0.623, *p* = 0.008) and VEmax (r= −0.509; *p* = 0.037). No significant correlations were found between 457-m PS time and IPE [BLa], HR max, RERmax, and IPE-RPE (*p* > 0.05).

## 4. Discussion

The present study found that a continuous graded intensity VO_2_max*_sw_* flume protocol demonstrated moderately strong test–retest reliability for both a health-fitness and competitive masters swimming sample of males and females. Results also established “performance validity” for swimming events of >6 min in duration. A standardized flume-based testing protocol can provide measures that, over time, reflect physiological adaptations of a prescriptive training program. Furthermore, our study’s results provide a robust application for those desiring to pursue a home-based swimming flume program (i.e., fitness, cross-training) in addition to those undertaking aerobic training for vocational purposes or competitive swimming events.

The ICCs ranged from r = 0.502 to 0.716 for VO_2_max_sw_, HRmax, VEmax, O_2_ pulse, and IPE [BLa]. These findings were somewhat lower than previous protocols that employed a self-regulated intensity strategy using both pool- and land-based settings [29,30,31]. Nevertheless, with the exception of IPE-RPE and RERmax, paired *t*-tests between Trials A and B for the physiological measures revealed no significant differences (*p* > 0.05), supporting the swimming flume protocol’s reproducibility. The ICC values from Stages 2 through 6 were statistically significant (ranging r = 0.465 to 0.669). The ICC for Stage 1 was not statistically significant. However, these results were not unexpected owing to wide variability and mode inter-individual differences in previous exposure and personal comfort at the beginning of the flume test protocol. When examining the overall test–retest reliability additional factors related to individual responses induced by the flume’s turbulent flow, as well as the protocol employed, must also be considered. The stage velocities were individualized for each participant, and intensity progression was intended to prevent excessive amounts of peripheral fatigue and/or disorientation associated with turbulence at greater flume velocities. Still, the overall findings should be considered statistically reproducible for the population tested.

Our findings also reveal the VO_2_max*_sw_* flume protocol demonstrated “performance validity” in health-fitness and masters competitive swimmers. The majority of participants had former age group and college competitive swimming experience. Therefore, these results may not be generalizable to a novice-level swimming population. Since maximal/peak physiological responses in water are comparatively different than land-based (i.e., cycle or treadmill) measures [13,18,30,31], an alternative paradigm was employed presently that utilized freestyle swimming performance time to establish “performance validity” as predicted by the swimming flume protocol. It was also expected that physiological responses to maximal swimming efforts in both the flume and pool were likewise controlled by the rate limiting links within the oxygen kinetic chain [30,31].

Results indicated that the current VO_2_max_sw_ flume protocol was a valid assessment of maximal aerobic power. This was demonstrated by a moderately strong R-value observed between VO_2_max_sw_ A (r = −0.648; *p* < 0.005), O_2_pulse (r = −0.623; *p* < 0.05), VEmax (r = −0.509; *p* < 0.05), and the 457-m performance swim. These results are more robust compared to previous studies using flume protocols and similar to VO_2_ measures obtained in pool settings [6,17,32,33,34]. The IPE-RPE, RERmax, and IPE [BLa] were not related to the 457-m performance swim (*p* > 0.05). However, using established physiological criteria, the mean values for RERmax (1.2) and IPE [BLa] (9.3 mmol∙L^−1^) confirmed a maximal effort at the point of test termination. Furthermore, the VO_2_max_sw_ Trial A and Trial B difference (1.21 mL∙kg^−1^∙min^−1^) was lower than established endpoint criteria (<2.1 mL∙kg^−1^∙min^−1^) for VO_2_max. In the present study, the secondary criteria, which reflected attainment of maximal aerobic power, were consistent with two previous studies of shallow water running and pool swimming [30,31].

For the majority of participants, the point of exhaustion occurred between the fifth and sixth flume stage. This proposes a predominant aerobic energy pathway contribution consistent with previous studies examining an association between aerobic power and swimming performances greater than 400 m [35]. While it appears that a greater capacity for oxygen transport and utilization plays a role in middle distance swimming performance, the present results do not fully explain all bioenergetic effects. Specifically, anaerobic energy contribution to a 400-m swim (>5 min) was shown to be as great as 20%, as well as additional anaerobic factors that include lactate dynamics [30,36]. Considering these factors, coaches should consider the inclusion of anaerobic or power training for middle and longer distances swimmers. The 457 m swimming criterion measure was also consistent with the experimental design of a larger overarching trial investigating military combat swimming performance. Regardless of the nature of the swimming event (i.e., vocational or competitive), future studies should also measure the metabolic demands for swimming bouts greater than 5 min in duration.

The regulatory functions of a swimming flume are to some degree standardized and should be viewed as analogous to a treadmill when used in a controlled laboratory environment. In the present study, the average time of flume test termination was greater than 7 min. The characteristics of the present study’s flume protocol allowed for systematic increases in velocity (aggressiveness and duration) similar to supramaximal paradigms that are shorter in duration and more aggressive. Such protocols have exhibited similar VO_2_max values compared to protocols with more conservative, graded, or ramped characteristics [31,37,38,39]. However, as a result of shorter test stages, identification of a metabolic steady state and precise individual swimming velocities by stage were not possible. Thus, swimming economy and corresponding energy cost could not be estimated for a given swimming stage. Still, the investigators recognize that the ability to externally regulate velocity using a swimming flume should expand beyond measuring VO_2_max_sw.._ The flume protocol would be advantageous to measure energy cost and swimming economy at submaximal velocities since it was shown to be a clear indicator of performance across a range of swimming distances [8]. Recent studies using poolside and flume protocols have identified critical velocity and measures of swimming economy as accurate determinants of swimming performance. Although not a priority focus of the present investigation, these measures can accurately predict aerobic capacity and may demonstrate a greater utility and ability to assist with training program design than VO_2_max*_sw_* alone [13,18,40,41,42].

The VO_2_max*_sw_* flume protocol and experimental design did present several limitations. Although participants practiced swimming in a stationary position at higher flume velocities, they occasionally became disoriented by water turbulence during near maximal intensity efforts of 90% and greater. Furthermore, measurement technicalities involving use of indirect calorimetry in a swimming flume environment should also be considered. Flow characteristics from a swimming flume are also known to change stroke mechanics in swimming, particularly upper body stroke parameters [19,43]. Although it appeared the suspended hose and metabolic mouthpiece did not impede the freestyle stroke, participants were occasionally unable to execute their natural swim stroke. Finally, it should be noted that constraints surrounding flume accessibility can be considered a drawback to this form of training.

In lieu of indirect calorimetry, a cost-effective approach to assessing swimmers in a flume would also involve accelerometry or other performance analysis metrics (i.e., wearable technology) in water that could assist with the development of statistical models to predict swimming performance [6,19,44,45]. Such specific models could identify characteristics of the swimming performance (i.e., open water, military training and operations, etc.) and also have application in a pool setting [46]. Exploring flume protocols that incorporate practical forms of technology applicable to a pool setting can assist with efforts to determine the energy cost associated with swimming performance and support new approaches to both testing and training. Furthermore, exploring this methodology in a swimming flume may provide application to pool performance swims, since similarities in measures of acceleration as a function of stroke mechanics (i.e., stroke rate and stroke count) were previously identified when comparing pool to flume swimming [19,47]. This study was not intended to elucidate the influence of stroke mechanics or fluid dynamics on the 457-m swim performance. However, investigating these variables could be particularly advantageous for the study of open water swimmers, triathletes, and military personnel since changes in velocity and fluid dynamics of a flume would simulate waves in open water conditions or close opponents during competition [7,48,49].

## 5. Conclusions

The continuous VO_2_max*_sw_* swimming flume protocol examined was found to have moderately strong test–retest reliability when measuring maximal oxygen uptake and provided a valid measure of the 457-m swim. The current flume protocol can be adopted as a valuable alternative method to pool protocols when evaluating aerobic fitness. Furthermore, with frequent assessments, an accurate swimming flume protocol can provide important feedback for those who train to improve health-related fitness and/or to enhance competitive performance.

## Figures and Tables

**Table 1:** Descriptive Characteristics (mean ± SD) of Participants.

Characteristic	Total (*n* = 19)
Age (yrs)	28.5 (8.3)
Height (cm)	174.7 (8.2)
Mass (kg)	72.9 (12.5)
BMI (kg·m^−2^)	23.7 (2.6)
Bodyfat (%)	21.4 (5.9)
Fat Free Mass (kg)	57.3 (11.8)
457 m swim (seconds)	469.4 (94.7)
VO_2_max*_sw_* (mL∙kg^−1^ ∙min^−1^)	46.7 (8.6)
Flume Stage Termination	5.7 (0.8)
Flume Time Termination (seconds)	477.76 (108.3)
HR max (beats·min^−1^)	172.0 (18.1)
Percent HR Max (%)	89.4 (9.0)
O_2_pulse (mL·beat^−1^)	0.3 (0.1)
RER Max	1.2 (0.3)
VEmax (L·min^−1^)	103.5 (20.2)
IPE-RPE (OMNI, 0–10)	8.5 (1.1)
IPE [BLa] (mmol·L^−1^)	9.3 (3.8)

**Table 2:** Test–Retest Reliability of VO_2_max*_sw_* Flume Protocol (*n* = 19).

Variable	VO_2_max*_sw_* Trial A	VO_2_max*_sw_* Trial B ^†^	ICC (95% CI)	*p*-Value	SEM	MD
VO_2_max*_sw_* (mL∙kg^−1^ ∙min^−1^)	46.7+/−8.6	47.9+/−8.5 ^^#^	0.628 (0.25, 0.83)	0.002	5.25	14.55
HRmax (beats∙min^−1^)	172.0+/−18.1	174.8+/−11.8 ^^#^	0.403 (−0.05,0.71)	0.041	11.86	32.87
O_2_pulse (mL·beat^−1^)	0.3+/−0.1	0.3+/−0.1 ^^#^	0.502 (0.06,0.77)	0.014	0.05	0.13
RERmax	1.2+/−0.3	1.1+/−0.2 ^^^	0.559 (0.16,0.80)	0.002	0.16	0.44
VEmax (L·min^−1^)	103.5+/−20.2	111.6+/−25.3 ^^#^	0.671 (0.32,0.85)	<0.001	12.49	34.62
IPE-RPE (OMNI, 0–10)	8.4+/−1.0	8.8+/−0.8 ^	0.539 (0.101,0.808)	0.006	0.58	1.59
IPE [BLa](mmol·L^−1^)	9.3+/−3.8	10.4 +/−2.5 ^^#^	0.716 (0.346,0.894)	<0.001	1.64	4.55
Final test time (s)	477.8+/−108.3	451.3 +/−54.5 ^^#^	0.608 (0.201,0.841)	0.004	52.66	145.97

VO_2_max*_sw_* values reported as mean ± *s*; ICC Values reported as r (95% CI). SEM—standard error of measurement; MD—minimal differences needed to be considered real. ^†^ Comparisons of VO_2_max*_sw_* Trial A and Trial B; # = indicates no significant paired *t*-test difference; ^ = *p* < 0.05 indicated significant test–retest reliability of Trial A vs. Trial B.

**Table 3:** Test–Retest Reliability of VO_2_ (mL∙kg^−1^∙min^−1^) By Flume Stage.

Test Protocol	VO_2_ Trial A	VO_2_ Trial B	ICC (95% CI)	*p*-Value	SEM	MD
Stage 1 (50% effort) (*n* = 17)	28.5+/−5.4	35.7+/−24.2	0.000 (−0.49, 0.42)	0.577	17.99	49.86
Stage 2 (70% effort) (*n* = 17)	31.6+/−5.9	31.3+/−5.2	0.469 (−0.01, 0.77)	0.029	4.11	11.39
Stage 3 (90% effort) (*n* = 17)	34.8+/−6.7	35.4+/−4.8	0.465 (−0.01,0.76)	0.030	4.32	11.98
Stage 4 (100% effort) (*n* = 17)	40.0+/−6.8	41.2+/−6.8	0.669 (0.30, 0.86)	0.001	3.92	10.85
Stage 5 (100% effort) (*n* = 15)	44.0+/−7.8	44.3+/−7.5	0.588 (0.13, 0.83)	0.008	5.00	13.86
Stage 6 (100% effort) (*n* = 9)	46.6+/−6.9	50.1+/−9.3	0.606 (0.01, 0.89)	0.014	4.35	12.05

VO_2_ values reported as mean +/− *s*; ICC values reported as r (95% CI). SEM—standard error of measurement. MD—minimal differences needed to be considered real.

**Table 4:** Performance Validity of VO_2_max*sw* Flume. Protocol: Trial A vs. Performance Swims.

Variable	457 m Swim Time
VO_2_max*_sw_* (mL∙kg^−1^ ∙min^−1^)	−0.648 (0.005)
HR max (beats∙min^−1^)	0.039 (0.881)
O_2_pulse (mL·beat^−1^)	−0.623 (0.008)
RERmax	−0.125 (0.633)
VEmax (L·min^−1^)	−0.509 (0.037)
IPE-RPE	0.322 (0.224)
IPE [BLa] (mmol·L^−1^)	−0.494 (0.061)

(*n* = 18; correlation coefficient (*p* value)).

## Data Availability

Data available on request due to restrictions around privacy. The data presented in this study are available on request from the corresponding author. The data are not publicly available due to the sensitive nature of the design and participants studied.
